# Naringenin’s Neuroprotective Effect on Diazino-Induced Cerebellar Damage in Male Albino Rats, with Modulation of Acetylcholinesterase

**DOI:** 10.3390/brainsci15030242

**Published:** 2025-02-25

**Authors:** Abdullah A. Saati

**Affiliations:** Department of Community Medicine and Pilgrims Healthcare, Faculty of Medicine, Umm Al-Qura University, Makkah 24382, Saudi Arabia; aaasaati@uqu.edu.sa

**Keywords:** naringenin, diazinon, neuroprotection, neuroinflammation, oxidative stress, AchE

## Abstract

Background: Diazinon, a well-known organophosphorus compound, is recognized for its neurotoxic effects, primarily through the inhibition of acetylcholinesterase (AChE) and induction of oxidative stress. Aim: This study evaluates the neuroprotective effects of naringenin, a citrus flavonoid, against diazinon-induced cerebellar damage in male albino rats. Materials and methods: Twenty-four rats were divided into four groups: control, naringenin, diazinon, and diazinon with naringenin. Results: Histological examination revealed altered structures of Purkinje cells in the cerebellum of the diazinon group. Naringenin co-treatment significantly improved cerebellar histology and modulated oxidative stress markers by decreasing malondialdehyde (MDA) and increasing glutathione (GSH) and glutathione peroxidase (GPx) levels. Additionally, naringenin exhibited anti-inflammatory effects by decreasing nuclear factor-kappa B (NF-κB), tumor necrosis factor-alpha (TNF-α), interleukin-6 (IL-6), and interleukin-1 beta (IL-1β) levels, while increasing interleukin-10 (IL-10). It also reduced apoptotic markers, including p53, Bax, caspase-9, caspase-8, and caspase-3, while increasing the anti-apoptotic marker Bcl-2. Furthermore, naringenin modulated AChE activity, leading to decreased acetylcholine levels and reduced neurotoxicity. Conclusions: These findings suggest that naringenin’s antioxidant, anti-inflammatory, and anti-apoptotic properties contribute to its neuroprotective role against diazinon-induced cerebellar damage.

## 1. Introduction

Diazinon is a well-known organophosphorus insecticide, with the inhibition of the acetylcholinesterase enzyme (AchE) considered key to its action [1]. Furthermore, it induces an imbalance between the production of free radicals and their elimination, resulting in cellular degeneration [1,2,3,4]. Several studies have demonstrated that diazinon reproduces multiple organ injuries [3,4,5,6]. Cognitive impairment, aggression, emotional changes, and neurotoxicity are considered complications of diazinon and organophosphorus exposure [7,8,9]. Poisoning with diazinon results in oxidative tissue damage due to an increase in oxidative stress markers [10]. It has been found that oxidative stress plays a crucial role in diazinon-induced neurotoxicity, in addition to its disruption of acetylcholine neurotransmission [11]. Oxidative stress results from an imbalance between the production of free radicals and the body’s ability to detoxify them. This imbalance leads to damage in lipids, proteins, and DNA, contributing to cellular dysfunction and death. In the case of diazinon-induced neurotoxicity, the excessive generation of reactive oxygen species (ROS) results in neuronal damage and cell death, further impairing cognitive and motor functions [12,13]. The cerebellum plays a pivotal role in coordinating motor control, balance, and cognitive functions. It is particularly vulnerable to oxidative stress and inflammation, making it a critical region to study in the context of diazinon-induced neurotoxicity. Previous studies have shown that cerebellar damage can significantly impair motor functions and cognitive abilities [14].

Despite the use of several antioxidants, such as vitamins E and C, N-acetyl cysteine, selenium, and natural compounds like safranal and crocin, against organophosphorus toxicities [15,16,17], there remains a critical need for more powerful protective agents to counteract the harmful effects of organophosphorus. Various flavonoids, such as quercetin, rutin, and luteolin, have shown promise in mitigating oxidative stress and inflammation in neurotoxic conditions.

Naringenin, a citrus flavonoid, has garnered attention for its neuroprotective properties. It exhibits several protective mechanisms against oxidative stress, inflammation, and apoptosis [18,19]. Various studies have documented the protective effects of naringenin against different neurotoxic agents, providing hope for its use against organophosphorus neurotoxicity [20,21]. For instance, naringenin has shown effectiveness in reducing oxidative stress and mitigating inflammation in models of neurotoxicity induced by other agents [22,23]. These studies highlight naringenin’s potential to protect neurons by scavenging free radicals, inhibiting pro-inflammatory cytokines, and preventing apoptotic cell death. To evaluate the neuroprotective effect of naringenin, we administered it to male albino rats exposed to diazinon and assessed its impact on cerebellar damage. We measured several parameters, including oxidative stress markers, inflammatory cytokines, and histopathological changes, to explore the various mechanisms through which naringenin exerts its protective effects.

## 2. Materials and Methods

### 2.1. Animals

Twenty-four male Wistar albino rats, aged 4 months and weighing 210–260 g, were involved in our research. These animals lived in an experimental environment with a 12-h light and 12-h dark cycle. During the study period, the rats were provided with unlimited fresh water and chow. The environmental temperature was maintained at 25 ± 1 °C. All our experimental studies followed the National Institutes of Laboratory Animal Care and Health guidelines (NIH publication no. 8023, which was revised in 1996).

### 2.2. Experimental Design

After a two-week acclimatization period, the animals involved in the study were classified into four groups (*n* = 6 for each group). The control group received 1.5 mL of 9% saline as a vehicle via oral gavage. The naringenin group received a dose of 100 mg/kg/day of naringenin orally, the selected dose according to previous work by Meng et al. [24]. The diazinon group received 25 mg/kg of diazinon orally, following the work of Ahmed et al. [25]. The diazinon + naringenin group received diazinon followed by naringenin one hour later, according to the previously mentioned doses. The study duration was fifteen days. On the last day, all rats were sacrificed through cervical dislocation. The cerebellum from each group was extracted and washed with ice-cold saline to remove blood. Some cerebellum samples were immersed in paraffin blocks for histological study, while others were stored at −80 °C for further biochemical and molecular assays.

### 2.3. Oxidative Stress Markers Evaluation

The cerebellar tissues were homogenized using a polytron homogenizer to prepare a 10% cerebellar homogenate at 4 °C in 0.05 M phosphate buffer. The homogenate was then centrifuged at 10,000 rpm (equivalent to 9391× *g*) for 20 min at 4 °C to produce the supernatant. All steps of sample preparation, including homogenization and centrifugation, were performed at 4 °C to prevent protein degradation and preserve enzymatic activity. The supernatant was stored at −80 °C for subsequent examinations. Malondialdehyde (MDA) levels were measured with commercial kits (CAT No. MD 2529) and detected by colorimetry at 534 nm, following Kei’s method [26]. Glutathione peroxidase (GPx) activity was assessed using kits (CAT No. GP2524) and a spectrophotometric method at 340 nm, based on Paglia and Valentine’s technique [27]. Reduced glutathione (GSH) activity was measured using kits (CAT No. GR 2511) and a colorimetric method at 405 nm, following Beutler et al.’s procedure [28]. All kits were provided by Biodiagnostic Co., Giza, Egypt.

### 2.4. Histopathological Examination

Cerebellar tissues were fixed overnight (approximately 12–16 h) in 4% paraformaldehyde at room temperature. After fixation, the tissues were dehydrated using an ascending gradient of alcohol: 70% ethanol for 1 h, 80% ethanol for 1 h, 90% ethanol for 1 h, 95% ethanol for 1 h, and 100% ethanol for 1 h (this step was repeated 3 times to ensure complete dehydration). The dehydrated tissues were embedded in paraffin wax and allowed to solidify. The paraffin blocks were sectioned at 4 µm thickness using a microtome. The sections were placed on glass slides and dried at 37 °C for 24 h to ensure proper adhesion of the tissue sections to the slides. For staining, the sections were stained with hematoxylin for 5 min, followed by rinsing in running tap water. The sections were then immersed in eosin for 2 min and rinsed again in running tap water. The stained sections were mounted using a permanent mounting medium Entellan and covered with coverslips. Finally, the cerebellar tissues were examined under a light microscope for histological evaluation [29]. To quantify the number of Purkinje cells in cerebellar sections, five H & E-stained sections were analyzed per rat per group. Five photomicrographs from each section were used, and the cells were counted in a 0.43 mm^2^ area using Image J software (Version 1.52f) with the integrated cell counter. The results were assessed at a magnification of ×400 [30].

### 2.5. Detection of AchE Activity, Acetylcholine Neurotransmitters Inflammatory, and Apoptotic Markers in the Cerebellar Supernatant

According to the method described by Ellman et al. [31], AchE activity in the cerebellar homogenate was assessed using an ELISA kit (Cat. No. CSB E11304r, Cusabio, Technology LLC., Houston, TX, USA). Additionally, acetylcholine cerebellar levels were measured using a kit (Catalog No. MBS8820077, MyBioSource, San Diego, CA, USA). Inflammatory markers, including nuclear factor-kappa beta (NF-κB-p65) (Catalog No. ELK1693, ELK Biotech, Wuhan, China), TNF-α, IL-6, IL-1β (Cat. Nos. CSB-E11987r, CSB-E04640r, CSB-E08055r-IS, Cusabio, Technology LLC., Houston, TX, USA), and IL-10 (Cat. No. ERA23RB, Thermo Fisher Scientific Inc., Waltham, MA, USA), were also measured. Apoptotic markers, such as Bax, caspase-9, caspase-3, Bcl-2 (Cat. Nos. CSB-EL002573RA, CSB-E08863r, CSB-E08857r, CSB-E08854r, Cusabio, Technology LLC., Houston, TX, USA), P53 (Cat. No. ERA47RB, Thermo Fisher Scientific Inc., Waltham, MA, USA), and caspase-8 (Catalog No. MBS260539, MyBioSource, San Diego, CA, USA), were also assessed. Briefly, cerebellar tissue was homogenized and centrifuged, and the supernatant was collected. A total of 100 µL of the supernatant was transferred to multiwell plates, kept at 37 °C for sixty minutes, and then removed. One hundred µL of biotin-antibody was then introduced and maintained at thirty-seven-degree Celsius for one hour, followed by five washes. A total of 90 microliters of TMB substrate reagent were incorporated, maintained for fifteen to thirty minutes at 37 °C, and then 50 µL of stop solution was introduced. The optical density was measured at 450 nm. Calibration standards were prepared to create a standard curve. Validation included running samples in duplicates or triplicates, using control samples, and ensuring consistent methods.

### 2.6. Statistical Analysis

Data were analyzed using GraphPad Prism 8.4.3, with statistical significance determined by one-way ANOVA followed by Tukey’s test (*p* < 0.05), and values were expressed as mean ± standard deviation.

## 3. Results

### 3.1. Ameliorative Effect of Naringenin Against Diazinon-Induced Cerebellar Damage

**Figure 1:**
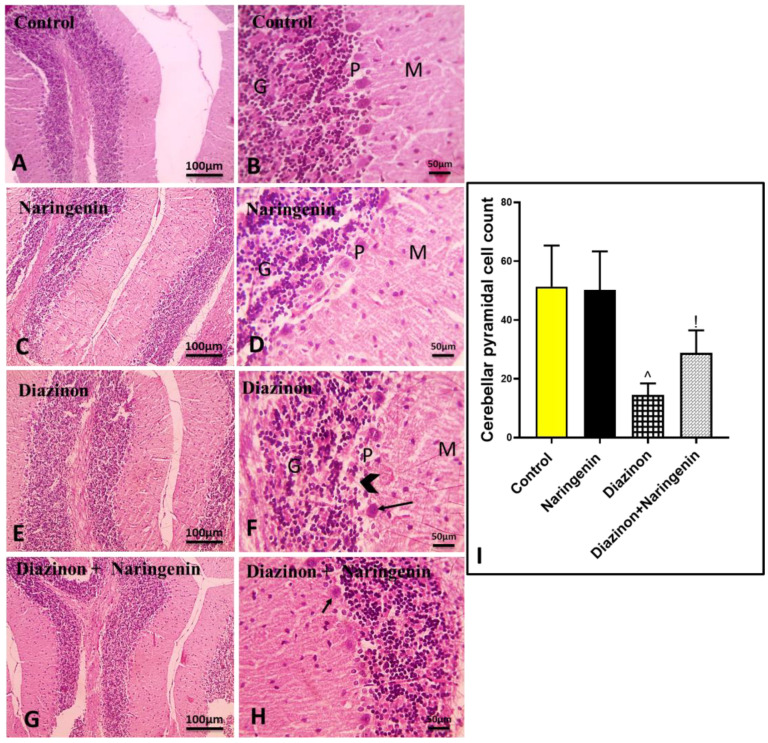
Microscopic images of H & E-stained cerebellar tissues. The control (**A**,**B**) and naringenin (**C**,**D**) groups show normal neurons in the grey matter, including the granular layer (GL), Purkinje layer (PL), and molecular layer (ML). The diazinon group (**E**,**F**) exhibits a decreased number of Purkinje neurons (arrowheads), with degeneration and necrosis in the remaining Purkinje neurons (black arrows). The diazinon + naringenin group (**G**,**H**) shows an increased number of normal flask-shaped Purkinje neurons (black arrows). (**I**) Pyramidal cell counting in different groups. Magnifications: ×100 (scale bar 100 µm) and ×400 (scale bar 50 µm). ^ *p* significant to control group, and ! *p* significant to the diazinon group (*p* < 0.05).

Histological examination of the hematoxylin-stained slides from all groups revealed that the control and naringenin groups exhibited normal cerebellar histology, with the three typical layers: granular, Purkinje, and molecular [Figure 1A–D]. In contrast, the slides from the diazinon group showed significant cerebellar damage, characterized by a marked decrease in the number of Purkinje neurons, with the remaining Purkinje cells appearing necrotic [Figure 1E,F]. Interestingly, the group treated with both diazinon and naringenin showed a notable increase in the number of healthy Purkinje cells [Figure 1G,H]. The ameliorative effect of naringenin on diazinon-induced cerebellar damage to Purkinje cells was confirmed by an increase in the number of Purkinje cells in histological scoring [Figure 1I].

### 3.2. Antioxidant Effects of Naringenin Against Diazinon-Induced Cerebellar Oxidative Damage

**Figure 2:**
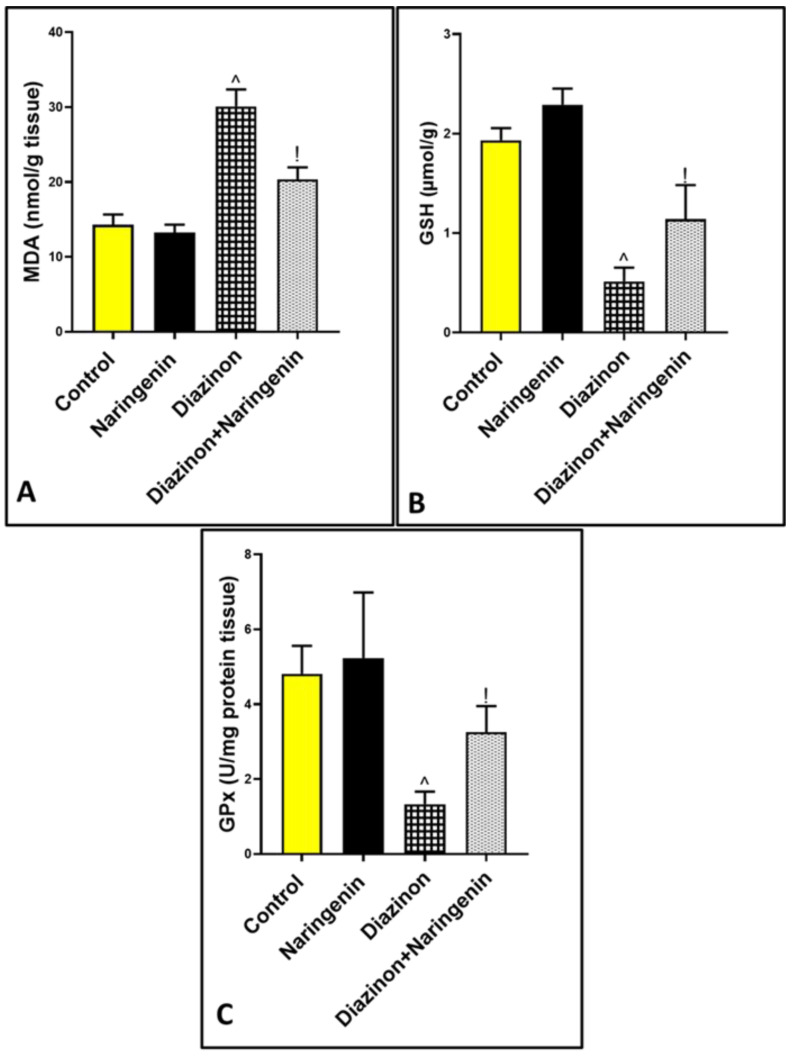
Graph representing the effects of diazinon and naringenin on oxidative stress markers: (**A**) MDA, (**B**) GSH, and (**C**) GPx in all studied groups. ^ *p* significant to control group, and ! *p* significant to the diazinon group (*p* < 0.05).

Oral intake of 25 mg/kg of diazinon resulted in a significant increase in MDA levels to 30.10 ± 2.72 compared to the control group at 14.32 ± 1.34 [Figure 2A]. This was accompanied by a notable decrease in the levels of reduced GSH and GPx activity in the cerebellar supernatant, with values of 0.51 ± 0.13 and 1.33 ± 0.33, respectively, compared to normal rats at 1.93 ± 0.12 and 4.81 ± 0.74 [Figure 2B,C]. However, the combination of diazinon with naringenin resulted in a marked decrease in MDA levels to 20.35 ± 1.60, and an increase in GSH and GPx levels to 1.14 ± 0.34 and 3.25 ± 0.69, respectively, compared to the diazinon group.

### 3.3. Anti-Inflammatory Role of Naringenin Against Diazinon-Induced Cerebellar Inflammation

**Figure 3:**
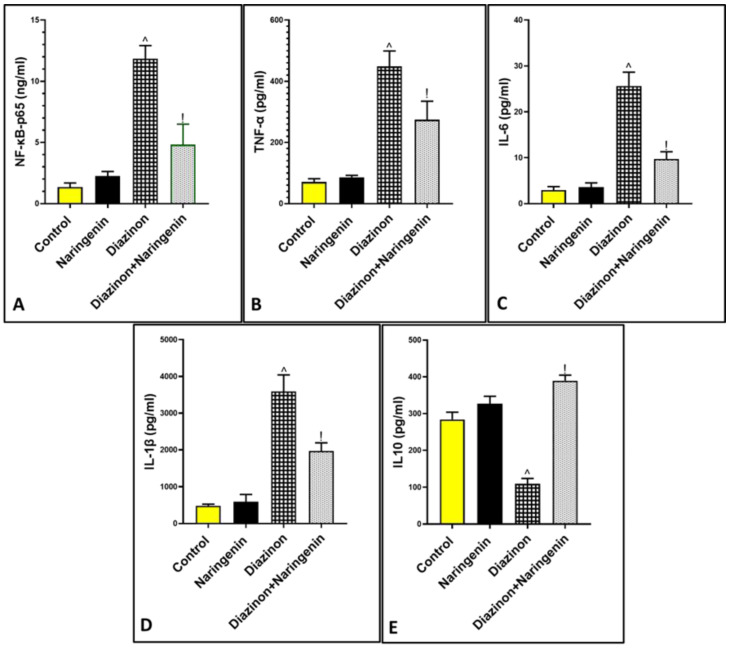
Effect of naringenin on inflammatory markers (**A**) NF-κB p65, (**B**) TNF-α, (**C**) IL-6, (**D**) IL-1β, and (**E**) IL10. ^ *p* means vs. control group, ! *p* vs. diazinon group.

As shown in Figure 3, the administration of diazinon significantly (*p* < 0.05) elevated the cerebellar protein level of the inflammatory transcription factor NF-κB to 11.85 ± 1.07, and the cytokines TNF-α, IL-6, and IL-1β to 448.8 ± 50.17, 25.68 ± 2.97, 3593 ± 448.6, respectively. However, it decreased the level of the anti-inflammatory IL-10 to 109.3 ± 14.83 in comparison to normal animals (1.35 ± 0.33, 70.83 ± 10.50, 2.94 ± 0.76, 482.8 ± 46.18, 284.3 ± 19.61, respectively). In contrast, oral intake of both diazinon and naringenin significantly mitigated neuroinflammation by decreasing inflammatory mediators to 4.83 ± 1.66, 274.2 ± 60.56, 9.72 ± 1.60, and 1971 ± 222, respectively, while increasing IL-10 levels to 389.7 ± 15.07. These findings suggest that naringenin possesses an anti-inflammatory effect against organophosphorus-induced cerebellar toxicity.

### 3.4. Anti-Apoptotic Effect of Naringenin Against Organophosphorus Diazinon-Induced Purkinje Apoptosis

**Figure 4:**
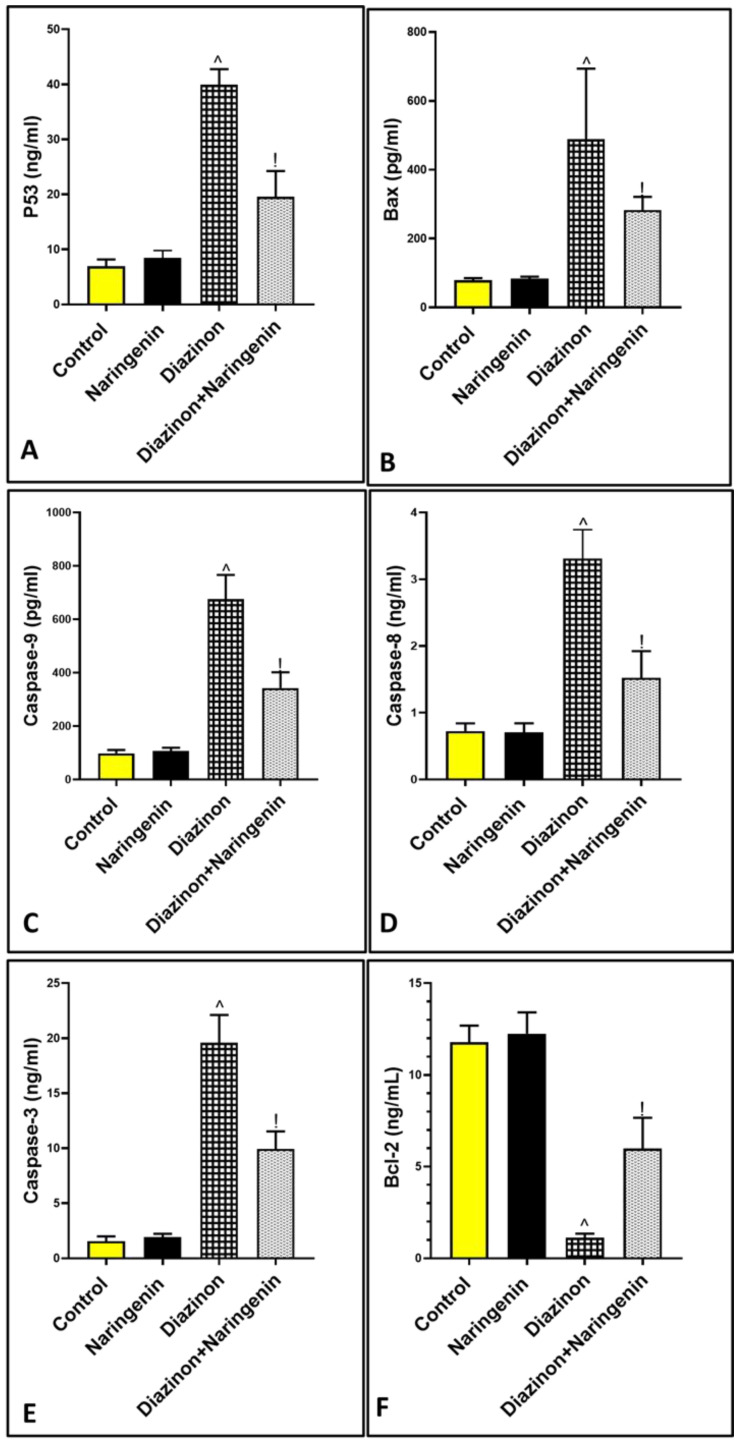
Graph illustrating the impact of diazinon and naringenin on apoptotic markers (**A**) P53, (**B**) Bax, (**C**) caspase-9, (**D**) caspase-8, (**E**) caspase-3, and (**F**) Bcl-2. ^ *p* means against normal rats, ! *p* versus diazinon-treated rats.

Administration of organophosphorus diazinon resulted in a significant elevation in the protein levels of the apoptotic markers P53, Bax, caspase-9, and caspase-8 (both extrinsic and intrinsic pathways), with a decrease in the anti-apoptotic marker Bcl-2. The levels were 39.93 ± 2.84, 488.7 ± 204.9, 675.7 ± 90.66, 3.31 ± 0.43, 19.58 ± 2.52, and 1.12 ± 0.21, respectively, compared to the control animals, which had levels of 6.95 ± 1.23, 78.5 ± 6.53, 97.83 ± 12.67, 0.72 ± 0.11, 1.55 ± 0.43, and 11.78 ± 0.90. Meanwhile, the combination of diazinon and naringenin significantly reduced Purkinje cell apoptosis by modulating the apoptotic and anti-apoptotic markers to 19.6 ± 4.64, 282.7 ± 38.42, 342.5 ± 59.38, 1.52 ± 0.39, 9.95 ± 1.59, and 5.98 ± 1.68, respectively [Figure 4A–F].

### 3.5. Excitatory Effect of Naringenin on AchE Activity in Rats Cerebellum

**Figure 5:**
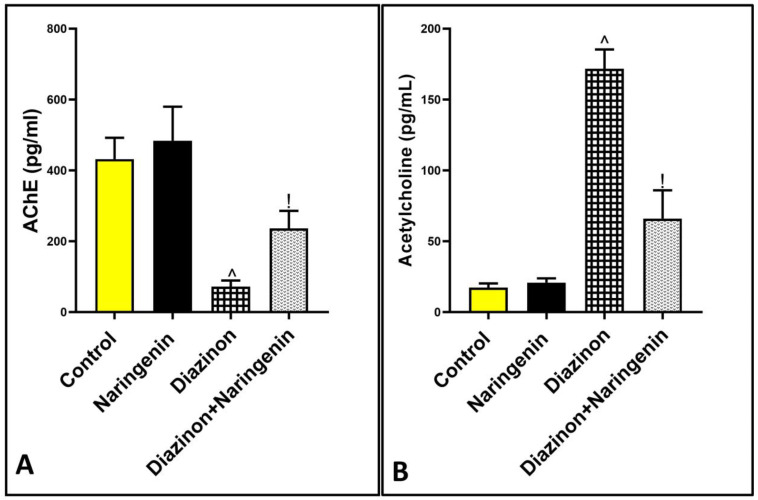
Effect of diazinon and naringenin on (**A**) AchE and (**B**) neurotransmitter acetylcholine in cerebellar tissues. ^ *p* means significance to normal group, ! *p* indicates significance to diazinon group.

Figure 5 shows a significant inhibition of AChE activity and an elevation in acetylcholine levels in the diazinon group, with values of 72 ± 17.37 and 171.8 ± 13.5, respectively, compared to the normal animals, which had values of 431.8 ± 60.56 and 17.33 ± 2.94. However, co-treatment with naringenin and diazinon reversed the AChE activity and acetylcholine levels to 236.7 ± 49.40 and 66 ± 19.95, respectively, in comparison to the diazinon group.

## 4. Discussion

Diazinon is a toxic organophosphorous pesticide that is harmful to humans [32,33]. The results of our study demonstrate the neuroprotective potential of naringenin against diazinon, as evidenced by the histological improvement in the number and structure of cerebellar Purkinje cells. This improvement can be attributed to naringenin’s antioxidant effect on cerebellar oxidative indicators, its inhibitory effect on inflammatory markers in cerebellar tissues, and its ability to decrease apoptotic markers in both intrinsic and extrinsic apoptotic pathways. Additionally, naringenin plays a key role in activating acetylcholinesterase activity, preventing the accumulation of acetylcholine, and thereby mitigating neurotoxicity and seizure formation.

The administration of organophosphorus compounds, including diazinon, produces marked oxidative stress, potentially due to their deregulatory effect on intrinsic antioxidant mechanisms [34,35]. Unfortunately, cerebellar tissues, as part of the brain, are particularly vulnerable to oxidative insults due to their high content of polyunsaturated fatty acids, which are prone to oxidation and lipid peroxide formation. Additionally, the cerebellum’s high oxygen consumption and low concentration of antioxidant enzymes further contribute to its susceptibility [36].

This fact was confirmed by the results of our study, which revealed that a 25 mg/kg oral intake of diazinon elevates MDA and depresses GSH and GPx. This is in line with previous work by Ezabadi et al. [10], who stated that diazinon intoxication produces an elevation of MDA and a depression of SOD, CAT, and GPx. Conversely, oral naringenin with diazinon produces a remarkable decrease in lipid peroxidation, with an enhancement in the activity of antioxidant enzymes in cerebellar tissues. This is concurrent with a previous study by Hassan et al. [16], who reported the ameliorative effect of naringenin against Alzheimer’s disease-induced cerebellar damage through its ability to halt oxidative stress by reducing MDA and NO while increasing cerebellar GSH. Additionally, a work by Md et al. [37] documented the neuroprotective effect of naringenin nanoparticles due to their antioxidant properties. Furthermore, a study by Rai et al. [38] discussed the antioxidant effect of naringenin against neuronal damage resulting from nanoparticulate aluminum through its elevation of antioxidant enzyme activity. The antioxidant effect of naringenin can be clarified by its power to scavenge free radicals and its inductive effect on endogenous antioxidant protective enzymes. Naringenin has been shown to possess potent antioxidant properties that mitigate oxidative stress by reducing lipid peroxidation and enhancing the activity of antioxidant enzymes. However, it is important to compare its effects with other well-known neuroprotective agents, such as curcumin, vitamin C, and N-acetyl cysteine (NAC), to provide a broader context. Curcumin, a compound found in turmeric, has demonstrated significant neuroprotective effects. Similar to naringenin, curcumin reduces oxidative stress by scavenging free radicals and enhancing the activity of endogenous antioxidant enzymes. Additionally, curcumin has been shown to attenuate neuroinflammation and apoptosis in various neurotoxicity models [39]. Vitamin C (ascorbic acid) is another potent antioxidant that protects neuronal cells from oxidative damage. Vitamin C neutralizes free radicals and regenerates other antioxidants such as vitamin E. Its neuroprotective role includes reducing lipid peroxidation and enhancing the levels of antioxidant enzymes, similar to naringenin [40]. NAC is a precursor to glutathione (GSH), one of the most important endogenous antioxidants. NAC enhances the synthesis of GSH, thereby protecting against oxidative stress. Its neuroprotective effects are attributed to its ability to reduce oxidative damage, decrease lipid peroxidation, and enhance the activity of various antioxidant enzymes [41]. The mechanisms by which naringenin, curcumin, vitamin C, and NAC exert their neuroprotective effects overlap significantly. All of these agents are known for their free radical scavenging abilities and their capacity to enhance the endogenous antioxidant defense system. The primary difference lies in their specific pathways and secondary effects. For example, curcumin also possesses anti-inflammatory properties, while NAC is particularly effective in replenishing intracellular GSH levels.

Neuroinflammation has been documented as one of the pathological mechanisms of organophosphorus involved in neuronal damage, and it has been found to be linked to oxidative stress [42]. Organophosphorus intoxication induces a drastic inflammatory reaction due to its stimulation of microglia and astrocytes, the primary immune cells in the CNS [43]. In the present study, cerebellar tissue examination with ELISA in the diazinon group revealed a significant rise in the inflammatory transcription factor NF-κB, and cytokines IL-6, IL-1β, and TNF-α, while there was a decrease in IL-10, which plays an anti-inflammatory role. This is in agreement with Afshari et al. [12], who reported that diazinon exposure elevates the level of TNF-α in the rat prefrontal cortex.

Fortunately, in this study, the fourth group of rats that received naringenin with diazinon showed a significant enhancement in the anti-inflammatory interleukin and a notable decrease in the inflammatory mediators in cerebellar tissues. This finding parallels the work done by Mansour et al. [21], who reported the neuroprotective and hepatoprotective roles of naringenin against lead acetate intoxication through its suppression of the inflammatory marker NF-κB and elevation of IL-10. Additionally, the study by Khajevand-Khazaei et al. [44] unveiled the protective character of naringenin against lipopolysaccharide-induced memory and learning impairments through its downregulation of TLR4, NF-κB, GFAP, COX2, iNOS, and TNF-α inflammatory markers. The anti-inflammatory role of IL-10 involves its ability to inhibit the synthesis of pro-inflammatory cytokines and reduce the activation of NF-κB [45]. Naringenin modulates these pathways by enhancing the production of IL-10, which in turn suppresses the inflammatory cascade triggered by NF-κB activation. This interaction highlights naringenin’s potential to attenuate inflammatory responses and protect neural tissues from oxidative stress and inflammation.

Apoptosis, also known as programmed cell death, can result from exposure to toxic substances. Many studies have explored the oxidative effects of diazinon, which triggers the apoptotic process in neuronal cells [46]. Additionally, neuroinflammation and oxidative stress resulting from diazinon toxicity led to neuronal apoptosis, primarily impacting the intrinsic apoptotic pathway due to mitochondrial dysfunction and cytochrome c release, resulting in neuronal deficits [47]. The results of our research showed a rise in the protein levels of both extrinsic and intrinsic apoptotic mechanisms in the cerebellar supernatant. Conversely, naringenin markedly reduced the ELISA levels of cerebellar apoptotic indicators. This finding parallels the work done by Mansour et al. [21], who documented that naringenin reduces apoptotic caspase-3 while increasing Bcl-2 in the cerebellum of lead acetate-treated rats, thereby decreasing apoptosis in Purkinje cells. Based on these findings, naringenin demonstrates a clear protective effect on Purkinje cells.

Diazinon is metabolized in the liver by cytochrome enzymes into diazinon oxon, its active analog [48,49]. This analog inhibits AChE, leading to diazinon-induced neurotoxicity. AChE is responsible for breaking down acetylcholine, a key cholinergic neurotransmitter. Inhibition of AChE results in the accumulation of acetylcholine, leading to cholinergic crises [50,51,52]. Our findings align with this, showing that diazinon significantly decreases AChE activity while increasing acetylcholine levels. However, co-treatment with naringenin modulates the levels of both AChE and acetylcholine. This is in line with the results of Heo et al. [53], who reported that naringenin inhibits AChE activity to prevent amnesia in Alzheimer’s disease. This discrepancy may be due to the toxic agent used in our study, an organophosphorus compound that inhibits AChE. Additionally, the difference may be attributed to the tissue examined, as our study focused on cerebellar tissue. It is important to note that naringenin elevated the level of AChE in comparison to the diazinon group but remained significantly lower than the levels observed in the control groups. This confirms the findings documented by Heo et al. [53]. In our study, we identified some limitations. We faced difficulties in performing behavioral examinations due to a lack of instruments, which was a result of limited resources. In future work, we aim to address this issue. Additionally, resource constraints prevented us from purchasing the primary antibodies needed to perform immunohistochemical examinations of certain markers, especially inflammatory and apoptotic ones. These analyses can be conducted in future studies.

## 5. Conclusions

In conclusion, naringenin demonstrates significant neuroprotective effects against diazinon-induced cerebellar damage. This protection is attributed to its potent antioxidant properties, anti-inflammatory effects, and ability to inhibit apoptosis. Additionally, naringenin enhances AchE activity, thereby mitigating neurotoxicity and preventing cholinergic crises. These findings suggest that naringenin could be a promising therapeutic agent for combating organophosphorus-induced neurotoxicity.

## Data Availability

The data that support this research will be shared upon reasonable request to the corresponding authors due to privacy restrictions.
